# The effects of physical exercise on cognitive function in adolescents: a systematic review and meta-analysis

**DOI:** 10.3389/fpsyg.2025.1556721

**Published:** 2025-07-28

**Authors:** Longyan Liu, Xianyang Xin, Ying Zhang

**Affiliations:** College of Physical Education and Training, Capital University of Physical Education and Sports, Beijing, China

**Keywords:** cognitive function, exercise interventions, adolescent, meta-analysis, RCT

## Abstract

**Objective:**

Physical exercise holds promise for improving cognitive function development in adolescents. However, current research evidence remains inconsistent. This systematic review and meta-analysis primarily aimed to determine the overall impact of physical exercise interventions on adolescent cognitive function. It also extended to explore effects on specific cognitive domains (such as executive function, attention, working memory, cognitive flexibility, and inhibitory control) and to examine potential differences across various exercise types.

**Method:**

A comprehensive search was conducted across multiple databases, including Web of Science, Embase, PubMed, the Cochrane Central Register of Controlled Trials, and CBM, to identify randomized controlled trials (RCTs) investigating the effects of physical exercise on cognitive function in adolescents. The search covered studies published from the inception of these databases through November 30, 2024. This protocol is registered with PROSPERO (CRD42024605962). A preliminary search of the databases and referenced citations identified 2,910 records. Standardized mean differences (SMD) and 90% confidence intervals (CI) were employed to calculate and aggregate the effect sizes for outcome measurement.

**Results:**

A total of 21 studies were included in this meta-analysis. Considering that cognitive function comprises multiple domains and that different studies use diverse outcome measures to evaluate adolescent cognitive function, a subgroup analysis was conducted. The studies were grouped into categories such as executive function, attention, working memory, cognitive flexibility, and inhibitory control to better capture changes in adolescent cognitive abilities. The findings demonstrate that physical exercise interventions lead to significant improvements in cognitive function among adolescents when compared to the control group. Notably, enhancements were observed in executive function (SMD = 0.21, 95% CI: 0.06 to 0.37), attention (SMD = 0.56, 95% CI: 0.34 to 0.78), cognitive flexibility (SMD = 0.42, 95% CI: 0.26 to 0.58), inhibitory control (SMD = 0.58, 95% CI: 0.22 to 0.94), and working memory (SMD = 0.54, 95% CI: 0.16 to 0.91). The subgroup analysis revealed that aerobic exercise had the greatest impact on cognitive function (SMD = 0.53, 95% CI: 0.32 to 0.73), particularly in areas such as executive function and attention, compared to other exercise modalities. This suggests that aerobic exercise may be particularly effective in enhancing adolescent cognitive abilities.

**Conclusion:**

Physical exercise has been shown to enhance cognitive function in adolescents. Based on the findings of this Meta-analysis, it is recommended that adolescents participate in at least moderate-intensity physical activities, such as aerobic exercise or resistance training, to promote cognitive development.

**Systematic review registration:**

https://www.crd.york.ac.uk/prospero/display_record.php?ID=CRD42024605962; Identifier: CRD42024605962.

## 1 Introduction

Cognitive function encompasses the fundamental mental abilities essential for performing daily activities, such as attention, memory, learning, perceptual-motor coordination, executive function, and language, among other skills (Bufano et al., 2024). Executive function (EF), a critical aspect of cognitive function, generally refers to “higher-level” cognitive abilities. It encompasses the control and regulation of “lower-level” cognitive processes, as well as goal-directed and future-oriented behaviors that are vital for navigating and achieving objectives (Alvarez and Emory, 2006). Additionally, it includes cognitive processes that organize and manage goal-directed actions, particularly in non-routine or novel situations (Banich, 2009). These functions empower us to comprehend complex or abstract concepts and tackle novel problems we have not previously encountered (Cristofori et al., 2019). Executive function is frequently a focus of physical exercise interventions, given its positive influence on various domains, including adolescent physical health (Miller, 2011), academic achievement, daily functioning (Bailey, 2007; Borella et al., 2010), as well as cognitive and psychological development (Diamond, 2009). Inhibitory control, cognitive flexibility, attention, and working memory constitute key components of cognitive function (Alvarez and Emory, 2006; Pennington and Ozonoff, 1996).

According to the World Health Organization (WHO) Global Action Plan for Adolescent Health 2023–2025, adolescence represents a pivotal stage for physical, psychological, and cognitive development. The maturation of cognitive abilities plays a crucial role in shaping adolescents’ academic performance, social competence, and overall future quality of life. However, changes in adolescent lifestyles have led to a rise in sedentary behaviors among today’s youth, including extended computer use and prolonged television viewing, which can adversely impact their cognitive function (Bidzan-Bluma and Lipowska, 2018). Paulich et al. (2021) analyzed data from the Adolescent Brain Cognitive Development (ABCD) Study, suggesting that increased screen time may impair children’s attention and impulse control—both critical components of cognitive function, and may potentially exacerbate the symptoms of Attention Deficit Hyperactivity Disorder (ADHD). Sleep plays a vital role in cognitive function, supporting emotional regulation, memory consolidation, and learning efficiency. Excessive screen time, however, may indirectly impair cognitive function by disrupting sleep quality (Barnett, 2008). Adolescent mental health issues, including anxiety, depression, and academic stress, are becoming increasingly severe on a global scale. These challenges significantly affect adolescents’ cognitive function and pose urgent public health concerns that require immediate and coordinated international attention.

However, physical exercise offers numerous benefits for the physical and mental health development of adolescents, with almost everyone benefiting from increased physical activity (Warburton and Bredin, 2017). These benefits include changes in adolescent body composition, fitness levels, and a range of health-related indicators, such as blood pressure, blood lipids, and insulin resistance (Janssen and LeBlanc, 2010). Compared to other interventions, physical exercise exerts a significant positive effect on brain development and cognitive function in adolescents, with notable improvements in attention, memory, and executive function. Zhang et al. (2024) explored the use of antipsychotic drugs (APs) to enhance adolescent cognitive function. However, evidence suggests that early administration of APs yields no significant positive effects on cognitive function and fails to effectively promote cognitive development. However, evidence suggests that acute exercise can significantly enhance cognitive function in adolescents across various domains, including attention (Budde et al., 2008), executive function (Costigan et al., 2017), working memory (Williams et al., 2022), cognitive flexibility (Silva et al., 2020), and inhibitory control (Leahy et al., 2020). These cognitive processes play a central role in self-regulation and goal-directed motor behavior (Cristofori et al., 2019), forming the foundation for effective learning. Research suggests that a primary mechanism through which physical exercise enhances cognitive ability is improved blood circulation within the cerebral cortex, particularly in the frontal lobe (prefrontal cortex), which plays a critical role in solving cognitively demanding tasks (Herold et al., 2018; Hillman et al., 2009). Furthermore, a single session of exercise can result in an immediate increase in cerebral blood flow associated with cognitive tasks following exercise cessation (Byun et al., 2014; Hyodo et al., 2012). However, studies have also reported cases of reduced cerebral blood flow or no observable effect (Moriarty et al., 2019). The variability in results may stem in part from differences in exercise duration, intensity, and timing, as well as the timing and methodology of cognitive assessments. Therefore, physical activity can enhance adolescents’ self-regulation abilities and emotional management skills, both of which contribute positively to the enhancement of cognitive function.

Although numerous studies have demonstrated that physical exercise can enhance cognitive function in adolescents (Donnelly et al., 2016; Hillman et al., 2009; Kubesch et al., 2009; Mazahery et al., 2019; Chen et al., 2017; Ren et al., 2024), the overall body of evidence remains inconsistent and reveals several critical gaps. For instance, while positive effects have been frequently reported, these findings are contradicted by other methodologically rigorous trials. Van Den Berg et al. (2018), for example, assessed cognitive performance before and after exercise interventions and reported no significant improvements in selective attention or working memory. Similarly, Pirrie and Lodewyk (2012) were unable to substantiate the hypothesis that physical activity improves cognitive outcomes in school-aged populations. In addition to these conflicting findings, the current literature is constrained by two major limitations. First, most existing studies have primarily focused on the effects of aerobic exercise—particularly on inhibitory control—while other key cognitive domains, such as working memory and cognitive flexibility, have received comparatively little attention (Ludyga et al., 2016). Second, previous systematic reviews (Chang et al., 2012; Etnier et al., 1997; Lambourne and Tomporowski, 2010; Sibley and Etnier, 2003) have largely emphasized the acute effects of exercise, leaving the cognitive implications of long-term or structured interventions insufficiently explored. As a result, the literature provides limited clarity regarding the differential impact of exercise modalities on executive function and its subcomponents, thereby underscoring the necessity of the present comprehensive meta-analysis.

Therefore, the primary aim of this systematic review and meta-analysis is to comprehensively examine and synthesize current evidence on the effects of various types of physical exercise interventions on different aspects of cognitive function in adolescents. Specifically, this study addresses the primary research question: What is the overall impact of physical exercise interventions on adolescent cognitive function? To further deepen our understanding, this review also explores secondary questions, including which specific cognitive domains (such as executive function, attention, working memory, cognitive flexibility, and inhibitory control) are most affected by exercise, and whether different exercise modalities (e.g., aerobic exercise, resistance training, high-intensity interval training, structured exercise, or game-based activity) produce differential effects.

## 2 Methods

This study was conducted following the Preferred Reporting Items for Systematic Reviews and Meta-Analyses (PRISMA) guidelines (Page et al., 2021). The review is registered with PROSPERO under the ID 2024-CRD42024605962.^1^

### 2.1 Search strategy

We conducted a comprehensive search of databases including Web of Science, Embase, PubMed, the Cochrane Central Register of Controlled Trials, and CBM, covering publications from the inception of each database up to November 2024. To ensure no relevant studies were omitted, we also manually reviewed the reference lists of the included studies and conducted a grey literature search. This study employed three categories of keywords: “cognition,” “adolescent,” and “exercise.” The first category encompassed terms such as “cognition,” “executive function,” “cognitive function,” and “cognitive ability.” The second category included keywords like “adolescent,” “youth,” “teen,” and “teenager.” The third category, related to exercise, consisted of terms such as “exercise,” “physical exercise,” “aerobic exercise,” “acute exercise,” “exercise training,” and “physical activity.” A search strategy combining subject terms and free terms was used, with the search query: (cognition OR executive function OR cognitive function OR cognitive ability) AND (Adolescent OR Youth OR Teen OR Teenager) AND (exercise OR physical exercise OR aerobic exercise OR acute exercise OR exercise training OR physical activity).

### 2.2 Inclusion and exclusion criteria

During the initial screening, all retrieved records were imported into reference management software (EndNote, version X9) to eliminate duplicate records. Three researchers (L.Y.L., X.Y.X., and Y.Z.) then independently screened the titles and abstracts to identify all potentially relevant studies, and any missing information was supplemented by contacting the authors. The inclusion of studies was based on the PICOS framework of evidence-based medicine, considering five main factors: participants, interventions, control groups, outcomes, and study design (Methley et al., 2014). A detailed summary of the eligibility criteria based on these components is provided in Table 1. This study included randomized controlled trials (RCTs) investigating the impact of physical exercise interventions on cognitive abilities and executive functions, including their subcomponents, in children and adolescents under the age of 18. The included studies were then independently screened by three researchers to identify all potentially relevant studies, with missing information being supplemented by contacting the authors. The result includes relevant indicators of executive function or cognitive function, as measured using any valid scale or test. Studies were excluded if they involved: (i) non-Chinese or non-English literature; (ii) non-interventional designs such as program reviews, cohort studies, case–control studies, reviews, systematic reviews, book chapters, or conference papers; or (iii) studies with missing or incomplete data.

**Table 1:** Eligibility criteria of the included studies.

Criterion	Description
Study Design	Randomized Controlled Trials (RCTs).
Participants	Adolescents under the age of 18 were included.
Intervention(s)	The intervention must involve physical exercise aimed at improving fitness, strength, coordination, or other physical aspects. Eligible forms include aerobic exercise, resistance training, high-intensity interval training (HIIT), structured exercise, and exercise combined with games. Studies using acute, short-term, or long-term physical activity were included.
Comparator/Control Group	Studies must include a control group. The control group could consist of participants receiving no intervention, engaging in placebo activities (e.g., non-exercise sedentary activities), or receiving usual activities/other non-physical exercise interventions.
Outcomes	Relevant indicators of cognitive function or executive function (and its subcomponents, such as attention, working memory, cognitive flexibility, inhibitory control) measured using valid scales or tests.
Setting/Location	Studies conducted in school, community, clinical, or other settings were eligible. No restrictions were applied to study setting.
Country	No geographic restrictions were applied. Studies from any country were included, provided they met the other inclusion criteria.
Date of Publication	Articles published from the inception of each database (Web of Science, Embase, PubMed, Cochrane Central Register of Controlled Trials, and CBM) through November 30, 2024, were included.
Language	Studies published in Chinese or English.
Publication Type	Peer-reviewed published articles. Exclusion criteria included program reviews, cohort studies, case–control studies, reviews, systematic reviews, book chapters, or conference papers.

### 2.3 Study selection and data extraction

The retrieved articles were independently double-blind screened by three researchers (L.Y.L., X.Y.X., and Y.Z.) according to the inclusion and exclusion criteria. The first step involved reading the titles and abstracts of the articles to exclude those that did not meet the inclusion criteria. The second step involved reading and screening the full texts of the remaining articles to determine the final studies for inclusion. The three researchers independently extracted relevant data from the included studies, including the following information: author names, publication year, participant characteristics (e.g., age and gender), number of participants in each group, intervention content, intervention duration, frequency, cycles, and reported outcomes. Data on the number of participants, mean values, and standard deviations (SD) for each group before and after the intervention were extracted from the studies included in the analysis.

### 2.4 Quality assessment

Bias risk assessment was conducted independently by two researchers (L.Y.L. and X.Y.X.) using the Cochrane Collaboration’s Risk of Bias Tool (Higgins et al., 2011). The assessment included random sequence generation, allocation concealment, blinding of both implementers and participants, blinding of outcome assessors, incomplete data, selective reporting, and other potential sources of bias. Any disagreements were resolved by a third arbitrator (Y.Z.).

### 2.5 Statistical analysis

The statistical analysis was performed using Review Manager 5.4 and Stata 16 software for effect size pooling, subgroup analysis, sensitivity analysis, and publication bias testing. As the measurements from baseline to endpoint for both the exercise and control groups were continuous variables, standardized mean differences (SMD) and 95% confidence intervals (CIs) were calculated based on the mean values, standard deviations, and sample sizes of the intervention and control groups. The I^2^ statistic was used to assess the heterogeneity between studies. I^2^ represents the heterogeneity of studies; when I^2^ ≤ 25%, it indicates no significant heterogeneity. When I^2^ ≤ 50% and I^2^ > 25%, the results are considered to have moderate heterogeneity. When I^2^ ≤ 75% and I^2^ > 50%, it indicates high heterogeneity. I^2^ indicates study heterogeneity; if I^2^ < 50% or *p* ≥ 0.05,a fixed-effects model was used to combine the effects; if I^2^ ≥ 50% and *p* < 0.05, a random-effects model was applied for analysis. Funnel plot symmetry was visually examined to assess publication bias. All tests were two-tailed, with a significance level set at α = 0.05; a *p*-value < 0.05 from the two-tailed tests was considered statistically significant. The forest plot was used to represent the statistical significance of the pooled effect size (SMD), where a small effect size is defined as SMD < 0.5, a medium effect size as 0.5 ≤ SMD < 0.8, and a large effect size as SMD ≥ 0.8. Egger’s test and funnel plots were used to conduct both quantitative and qualitative analyses of publication bias in the included studies.

## 3 Results

### 3.1 Search results

**Figure 1:**
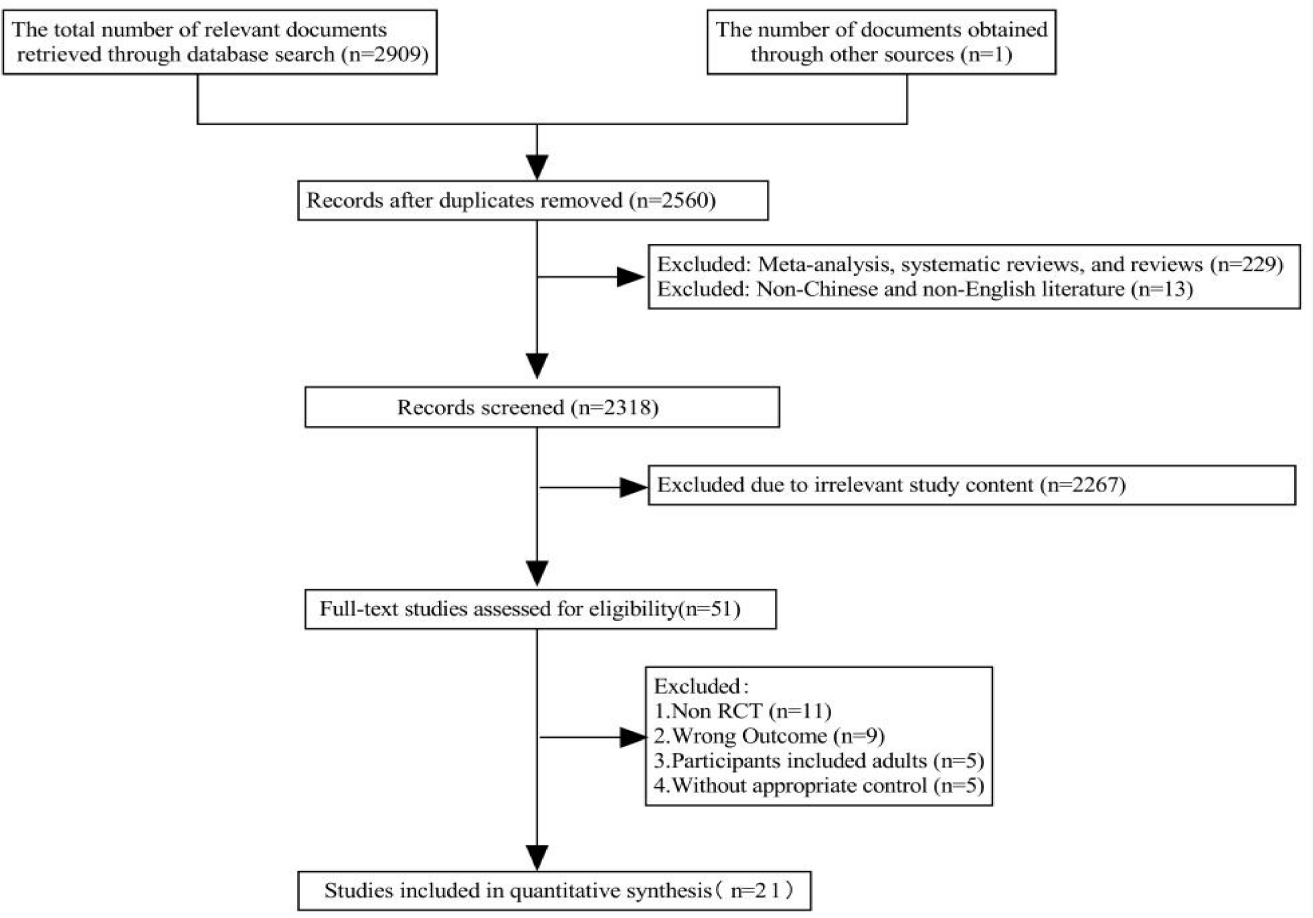
Study flow diagram.

A total of 2,909 articles were retrieved from the database search, along with one additional article identified through reference screening, resulting in an initial pool of 2,910 articles. After removing 350 duplicate records, 2,560 studies remained for the initial screening of titles and abstracts. Of these, 51 studies were selected for full-text review, and ultimately, 21 articles were included in the final analysis (Figure 1).

### 3.2 Basic characteristics of included studies

A total of 21 RCT studies were included in the meta-analysis. The included studies were predominantly published in the past 20 years, with a total of 3,544 participants. Participants were divided into two groups: the control group (1814 participants) and the experimental group (1730 participants). The duration of the exercise interventions, reported as outcome measures, ranged from 2 week to 39 weeks, with each study having its own intervention duration and frequency (Table 2).

**Table 2:** Overview of basic characteristics of included literature in meta-analysis.

Included Studies	Country	Research type	Age (Years)	Participants (M/F)	Sample Size (*N*)	Intervention characteristics	Intervention Duration (Weeks)	Session Duration	Session Frequency	Outcome measures
Altermann and Gröpel (2023)	Austria	RCT	16.5 ± 1.12	80 (39/41)	IG = 60; CG = 20	25-min high-intensity interval training (HIIT)	/	25 min	/	Attention:(D2-test)
						25-min full-body strength circuit workout	/	25 min	/	Attention:(D2-test)
						25-min coordination circuit training	/	25 min	/	Attention:(D2-test)
Costigan et al. (2017)	Australia	RCT	15.8 ± 0.6	65 (45/20)	IG = 43; CG = 22	Aerobic Exercise Program: HIIT sessions(e.g., shuttle runs, jumping jacks, skipping)	8	8-10 min	3 times/week	executive function:(TMT)
						Resistance and Aerobic Program: HIIT sessions (e.g., shuttle runs, jumping jacks, skipping, combined with body weight squats, push-ups)	8	8-10 min	3 times/week	executive function:(TMT)
Benzing et al. (2016)	Switzerland	RCT	14.52 ± 1.03	42 (42/0)	IG = 21; CG = 21	“Shape Up” game: aerobics	/	15 min	/	inhibitory control:(D-KEFS)
						“run the world”game:run	/	15 min	/	Cognitive flexibility:(D-KEFS)
Williams et al. (2022)	United Kingdom	RCT	11.7 ± 0.3	16 (0/16)	IG = 8; CG = 8	sprint training	2	Week one:5.5 min Week two:6.5 min	3 times/week	Working memory:(Sternberg test) executive function:(Stroop test) inhibitory control:(Flanker task)
Altenburg et al. (2016)	Netherlands	RCT	11.4 ± 0.8	36	IG = 17; CG = 19	dance activities	/	20 min	/	Attention:(TEA-Ch)
Budde et al. (2008)	Germany	RCT	14.98 ± 0.78	99 (80/19)	IG = 47; CG = 52	Acute coordinative exercise	2	7.5 min	2 times/week	Attention:(D2-test)
Ranjani et al. (2023)	India	RCT	/	2000	IG = 996; CG = 1,004	yoga	17	35 min	2 times/week	Attention:(LCT)
Subramanian, 2015	India	RCT	14 ± 1	275 (152/123)	IG = 136; CG = 139	Structured Physical activity	26	120 min	6 times/week	Attention:(LCT) executive function:(TMT) cognitive flexibility:(RFFT)
Hillman et al. (2014)	United States	RCT	/	221 (119/102)	IG = 109; CG = 112	Aerobic fitness	21	70 min	/	inhibitory control:(Flanker task) cognitive flexibility:(Switch task)
Kamijo et al. (2011)	United States	RCT	9.1 ± 0.6	36 (17/19)	IG = 20; CG = 16	physical fitness exercise	39	120 min	2 times/week	Working memory:(Sternberg task)
Ludyga et al. (2018)	Switzerland	RCT	12.5 ± 0.7	36 (23/13)	IG = 20; CG = 16	aerobic and coordinative exercises	8	20 min	5 times/week	inhibitory control:(Stroop task)
Kim and So (2015)	Korea	RCT	9.20 ± 1.40	20 (8/12)	IG = 10; CG = 10	resistance and aerobic exercise programmed	16	90 min	2 times/week	inhibitory control:(Stroop task)
Silva et al. (2020)	Brazil	RCT	12 ± 2	20 (14/6)	IG = 10; CG = 10	swimming–learning program	8	45 min	2 times/week	cognitive flexibility:(TMT) Attention:(TAC)
Leahy et al. (2020)	Australia	RCT	16.2 ± 0.4	62 (32/30)	IG = 33; CG = 29	HIIT sessions	14	12-20 min	3 times/week	inhibitory control:(Flanker task) Working memory:(n-back task)
Davis et al. (2011)	United States	RCT	9.3 ± 1.0	116	IG = 56; CG = 60	Aerobic exercise	13	40 min	/	executive function:(CAS)
Davis et al. (2007)	United States	RCT	9.2 ± 0.84	61	IG = 32; CG = 29	Aerobic fitness exercise	15	40 min	5 times/week	executive function:(CAS)
Krafft et al., 2014	United States	RCT	9.9 ± 0.9	43 (15/28)	IG = 19; CG = 24	Aerobic exercise	34	40 min	5 times/week	inhibitory control:(Flanker task)
Chou et al. (2023)	China	RCT	11 ± 0.64	50	IG = 25; CG = 25	competitive team games	10	40 min	5 times/week	inhibitory control:(Flanker task)
Alvarez and Emory (2006); Chou et al. (2019)	China	RCT	12.19 ± 0.68	84 (52/32)	IG = 44; CG = 40	Movement Games	8	40 min	3 times/week	inhibitory control:(Stroop test)
Chen et al. (2016)	China	RCT	12.64 ± 70	50 (28/22)	IG = 25; CG = 25	Aerobic exercise	13	40 min	4 times/week	Executive function:(WCST)
Gallotta et al. (2015)	Italy	RCT	/	152	IG = 83; CG = 69	Coordinative exercises	21	60 min	2 times/week	Attention:(d2-test)

M, man; W, woman; IG, intervention group; CG, control group; D2-test, D2-test of attention; MT, The Trail Making Test; D-KEFS, Delis-Kaplan Executive Function System; TEA-Ch, Test of Selective Attention in Children; LCT, Letter Cancellation Test; RFFT, Ruff Figural Fluency Test; TAC, TAC Cancellation Attention Test; CAS, the Cognitive Assessment System; WCST, Wisconsin Card Sorting Test.

### 3.3 Risk of bias assessment

**Figure 2:**
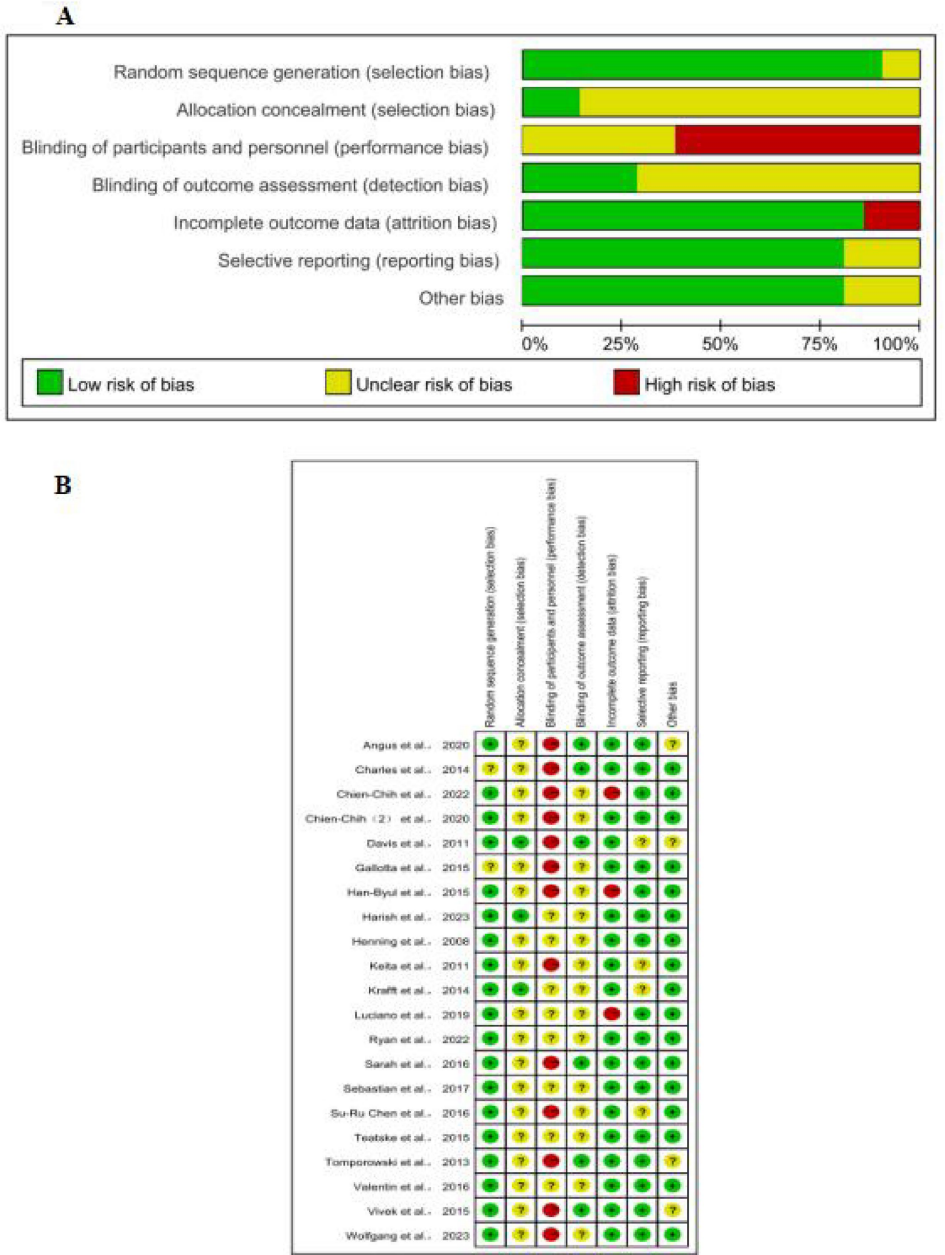
**(A)** Risk of bias summary; **(B)** risk of bias assessments.

The methodological quality of the 21 included studies, summarized in Figure 2, varied across the assessed domains. A majority of studies demonstrated a low risk of bias in random sequence generation and selective reporting. However, significant concerns were identified in other areas. The risk related to allocation concealment was frequently unclear. A high risk of performance bias was noted across nearly all studies, reflecting the inherent challenge of blinding participants and personnel in exercise interventions. Additionally, several studies presented a high risk of attrition bias due to dropout rates. In contrast, the blinding of outcome assessment was generally well-conducted, presenting a low risk of detection bias in most trials. Overall, while the included studies possess methodological strengths, the notable risks related to performance bias and attrition bias warrant consideration when interpreting the findings of this meta-analysis (Figure 2).

### 3.4 Results of meta-analysis

#### 3.4.1 The intervention effects of physical exercise on cognitive function in adolescents

The effects of exercise interventions on executive function and its subcomponents were reported as mean ± standard deviation (SD). For measures where a smaller mean reflects better performance (e.g., shorter response time or fewer errors), the mean value was converted to an absolute value. As a result, a positive means consistently represents an improvement in cognitive performance.

##### 3.4.1.1 Executive function

**Figure 3:**
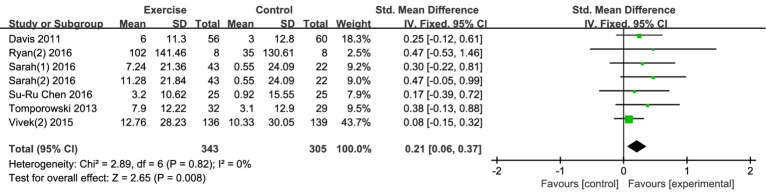
Forest plot of executive function.

Seven studies examined the effects of exercise interventions on adolescent executive function. The findings demonstrated that exercise interventions positively influenced adolescent executive function (Figure 3). Compared to the control group, the exercise intervention led to an improvement in executive function, with [SMD = 0.21, 95% CI (0.06, 0.37), *p* = 0.008]. Notably, there was low heterogeneity among the studies (I^2^ = 0%), indicating a high degree of consistency in the results. While the effect size is small, it still suggests that incorporating exercise interventions into educational or developmental programs could have meaningful benefits for improving executive function in adolescents. Given that executive function includes key cognitive skills such as attention, planning, and decision-making, even a small improvement in these areas could have a positive impact on academic performance, behavior regulation, and overall life skills. The low heterogeneity (I^2^ = 0%) indicates that the results were consistent across the seven studies, further supporting the reliability of the findings. This suggests that exercise interventions might provide a robust, generalizable benefit to adolescents’ cognitive function, with consistent positive effects across different populations and study designs.

##### 3.4.1.2 Attention

**Figure 4:**
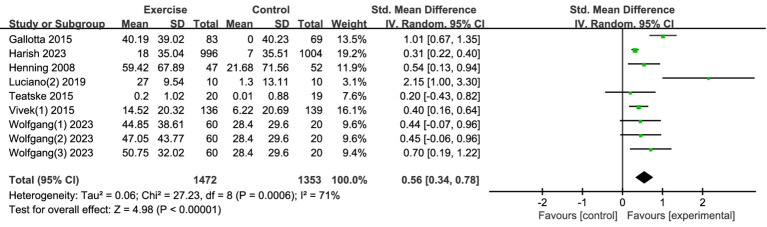
Forest plot of attention.

Nine studies examined the effects of exercise interventions on adolescent attention. The findings demonstrated that exercise interventions significantly improved adolescent attention (Figure 4). Compared to the control group, the exercise group exhibited notably higher attention scores [SMD = 0.56, 95% CI (0.34, 0.78), *p* < 0.00001], indicating a clear advantage of exercise interventions over the control group. However, substantial heterogeneity was observed among the studies (I^2^ = 71%), reflecting considerable variability in the results. Consequently, a random-effects model was applied for Meta-analysis. The moderate to large effect size suggests that exercise interventions can have a considerable impact on adolescents’ attention. Given that attention is a foundational cognitive skill that influences learning, academic performance, and daily functioning, improving attention through exercise could contribute to better school performance, more effective learning strategies, and improved concentration in various settings. This effect is particularly significant because attention is critical for tasks requiring sustained focus, such as studying or working.

##### 3.4.1.3 Cognitive flexibility

**Figure 5:**
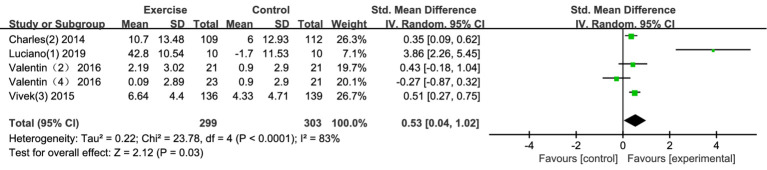
Forest plot of cognitive flexibility.

Five studies examined the effects of exercise interventions on adolescent cognitive flexibility. The findings demonstrated that exercise interventions had a positive effect on cognitive flexibility (Figure 5). Compared to the control group, the exercise interventions had a significantly greater impact on cognitive flexibility [SMD = 0.42, 95% CI (0.26, 0.58), *p* < 0.00001]. However, there was substantial heterogeneity among the studies (I^2^ = 83%), reflecting significant variability in the results. Sensitivity analysis identified a study by Silva et al. (2020) as having a notable impact on the overall results. After excluding this study, heterogeneity decreased to a moderate level (I^2^ = 48%, *p* = 0.12), and the pooled effect remained significant (SMD = 0.33, 95% CI [0.07, 0.59], *p* = 0.01), suggesting that the results were robust. The small-to-moderate effect size indicates that exercise can have a meaningful impact on cognitive flexibility, a crucial skill that allows individuals to adapt to changing demands, switch between tasks, and think creatively. Enhancing cognitive flexibility can improve problem-solving, adaptability, and overall mental flexibility, which are valuable skills for adolescents in both academic and real-life contexts.

##### 3.4.1.4 Inhibition control

**Figure 6:**
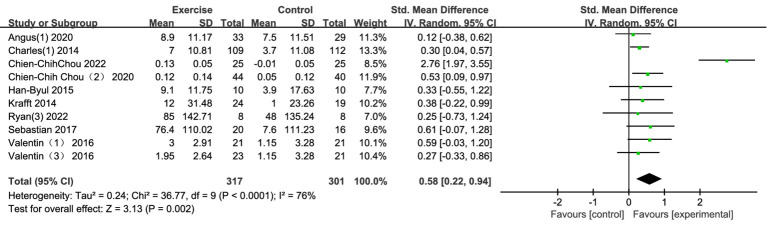
Forest plot of inhibition control.

Ten studies examined the effects of exercise interventions on adolescent inhibitory control. The findings demonstrated that exercise interventions had a positive impact on inhibitory control in adolescents (Figure 6). The initial meta-analysis of the 10 studies revealed a significant pooled effect [SMD = 0.58, 95% CI (0.22, 0.94), *p* = 0.002]; however, substantial heterogeneity was observed among the studies (I^2^ = 76%). To investigate the source of this heterogeneity, a sensitivity analysis was conducted. The analysis identified that the study by Chou et al. (2019) was the primary source of heterogeneity. After excluding this study, the heterogeneity was eliminated (I^2^ = 0%, *p* = 0.94), and the pooled effect size for inhibitory control remained significant and robust (SMD = 0.36, 95% CI [0.19, 0.52], *p* < 0.0001). This robust small-to-moderate effect size indicates that exercise interventions have a meaningful impact on inhibitory control, a critical aspect of executive function. Inhibitory control is essential for regulating behavior, controlling impulses, and resisting distractions, which are key for academic success, social interactions, and personal development. Improving inhibitory control through exercise could help adolescents better manage their emotions, behavior, and decision-making processes in everyday life.

##### 3.4.1.5 Working memory

**Figure 7:**
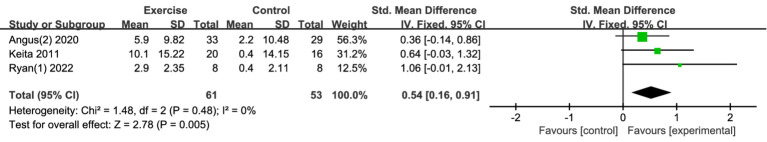
Forest plot of working memory.

Three studies examined the effects of exercise interventions on adolescent working memory. The findings demonstrated that exercise interventions positively influenced working memory in adolescents (Figure 7). Compared to the control group, exercise interventions led to significant improvements in working memory, with [SMD = 0.54, 95% CI (0.16, 0.91), *p* = 0.005], highlighting the superior impact of exercise interventions over the control group. No significant heterogeneity was observed among the studies (I^2^ = 0%), indicating a high level of consistency in the results. The moderate effect size indicates that exercise interventions can significantly improve working memory, a vital cognitive function that is essential for tasks involving mental calculation, learning, and problem-solving. Improving working memory through exercise can have important implications for academic performance, as working memory is crucial for retaining and manipulating information. A small to moderate improvement in working memory could lead to better academic outcomes and cognitive functioning in everyday activities.

#### 3.4.2 The results of the subgroup analysis

**Figure 8:**
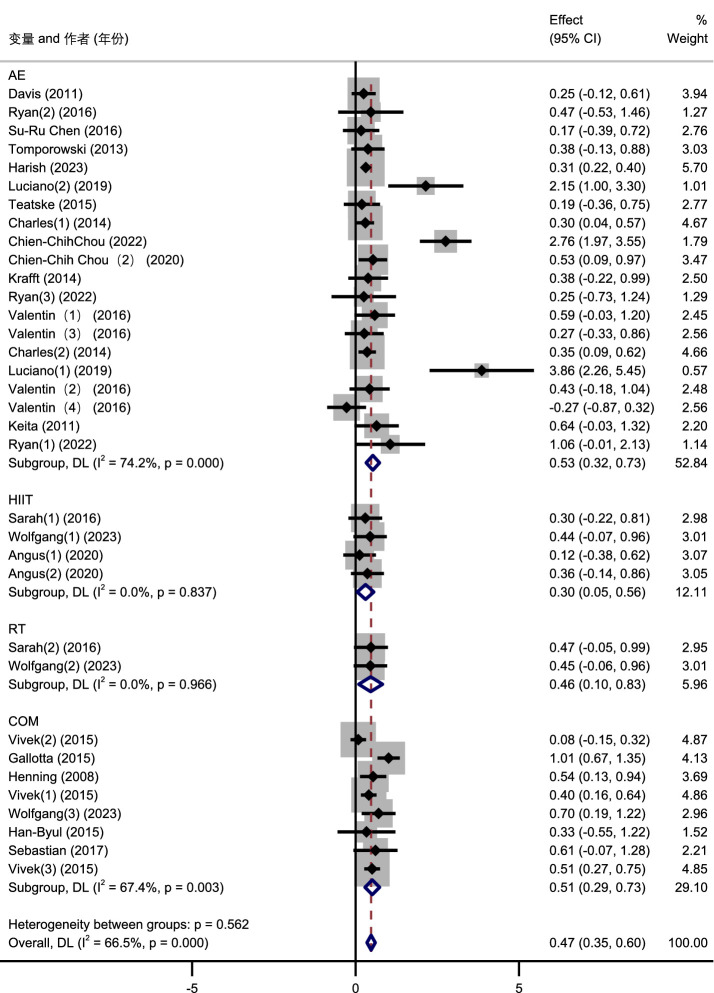
Subgroup analysis forest plot of the impact of physical exercise on adolescents’ cognitive function.

In the subgroup analysis based on exercise types, aerobic exercise (AE) showed a significant positive effect on cognitive function in adolescents, with a standardized mean difference (SMD) of 0.53 (95% CI: 0.32 to 0.73; I^2^ = 74.2%). High-intensity interval training (HIIT) also demonstrated a significant improvement, with an SMD of 0.46 (95% CI: 0.08 to 0.83; I^2^ = 0.0%). Resistance training (RT) had a comparable positive effect, with an SMD of 0.48 (95% CI: 0.10 to 0.86; I^2^ = 0.0%). Combined exercise (COM) interventions yielded an SMD of 0.46 (95% CI: 0.18 to 0.75; I^2^ = 67.4%), indicating moderate heterogeneity. Overall, the pooled effect size across all studies was 0.47 (95% CI: 0.35 to 0.60; I^2^ = 66.5%). No significant differences were found between subgroups (*p* = 0.562), suggesting that various types of physical exercise are similarly effective in enhancing cognitive function among adolescents (Figure 8).

### 3.5 Publication bias

**Figure 9:**
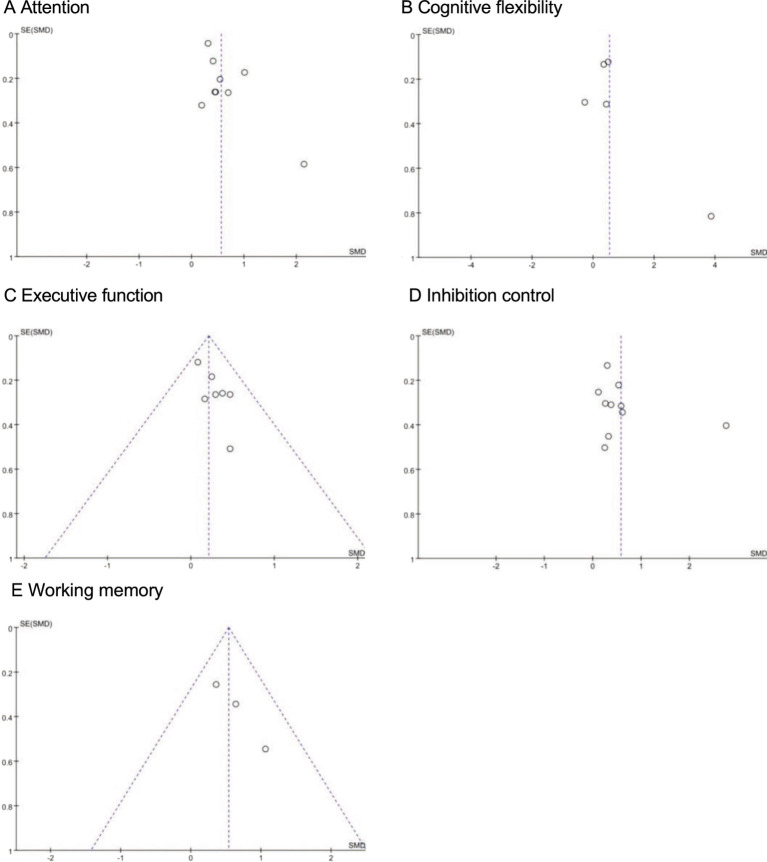
Funnel plot assessing the publication bias.

Egger’s test revealed varying degrees of publication bias risk across the cognitive domains included in this meta-analysis. For attention (t = 2.88, *p* = 0.027), a significant small-sample effect was detected, indicating potential publication bias. The marginal significance of the *p*-value (t = 2.35, *p* = 0.051) further suggests a moderate risk of bias. For cognitive flexibility (t = 0.30, *p* = 0.783; *p* = 0.498), no significant small-sample effect or publication bias was observed, suggesting a low risk of bias. For executive function (t = −0.51, *p* = 0.634), no small-sample effect was detected; however, significant publication bias (t = 3.02, *p* = 0.029) indicates a moderate to high bias risk. For inhibitory control (t = 0.02, *p* = 0.985; *p* = 0.284), no small-sample effect or publication bias was identified, indicating a low bias risk. Similarly, for working memory (t = −2.34, *p* = 0.257; *p* = 0.085), no significant publication bias was detected, but the marginal significance of the *p*-value suggests that caution is warranted when interpreting the results. Overall, the findings indicate a moderate to high risk of publication bias in the attention and executive function domains, whereas the bias risk for cognitive flexibility, inhibitory control, and working memory is relatively low. These results underscore the importance of future research incorporating larger sample sizes, standardized methodologies, and bias correction techniques to improve the reliability of conclusions (Figure 9)

### 3.6 Sensitivity analysis

**Figure 10:**
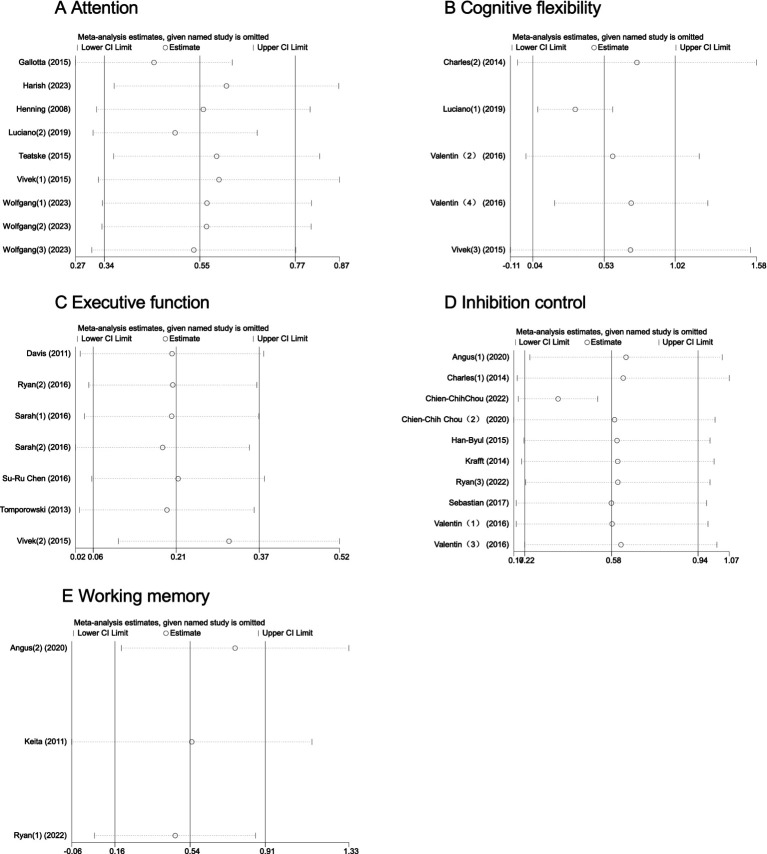
Sensitivity analysis of the impact of physical exercise on adolescents’ cognitive function.

To ensure the reliability of the meta-analysis results, we conducted sensitivity analyses to examine the impact of each study on the overall effect. By sequentially removing each study, we assessed its influence on the overall effect size and heterogeneity. The results showed that, after excluding any single study, the overall effect size and confidence intervals changed minimally, with good consistency across studies, indicating that the meta-analysis results are stable and reliable (Figure 10).

## 4 Discussion

This study examines the effects of physical exercise interventions on cognitive function in children and adolescents from an evidence-based medicine perspective. By analyzing the outcomes of various physical interventions and cognitive function tests across multiple randomized controlled trials involving diverse groups of children and adolescents, the study aims to provide a deeper understanding of these effects.

The findings indicate that physical exercise interventions can significantly enhance cognitive function in children and adolescents. Specifically, the results on inhibitory control reveal that physical exercise exerts the most substantial positive effect on inhibitory control abilities [SMD = 0.58, 95% CI (0.22, 0.94), *p* = 0.002], with improvements observed across all groups. This result aligns with previous meta-analytic findings (Verburgh et al., 2014), which may be attributed to inhibitory control being more susceptible to the influence of physical exercise compared to other aspects of executive function (Tomporowski et al., 2008).

The findings of this study demonstrate that physical exercise significantly enhances cognitive performance in children and adolescents across multiple domains, including executive function [SMD = 0.21, 95% CI (0.06, 0.37), *p* = 0.008], attention [SMD = 0.56, 95% CI (0.34, 0.78), *p* < 0.00001], cognitive flexibility [SMD = 0.42, 95% CI (0.26, 0.58), *p* < 0.00001], working memory [SMD = 0.54, 95% CI (0.16, 0.91), *p* = 0.005], and inhibitory control [SMD = 0.58, 95% CI (0.22, 0.94), *p* = 0.002]. This finding aligns with previous Meta-analyses, which indicate that physical exercise has a smaller effect on children with or without learning or physical disabilities (d = 0.32) (Page et al., 2021; Verburgh et al., 2014), whereas the overall effect on typically developing individuals is moderate (d = 0.52) (Verburgh et al., 2014).

A noteworthy finding of this meta-analysis was the substantial initial heterogeneity observed for the inhibitory control (I2 = 76%) and cognitive flexibility (I2 = 83%) outcomes, which prompted further investigation into potential sources of this variability. For inhibitory control, the sensitivity analysis revealed that the heterogeneity was primarily attributable to the study by Chou et al. (2023) which reported a considerably larger effect size than other trials. This discrepancy may be explained by the specific nature of their intervention—'cognitively engaging movement games’. It is plausible that such enriched interventions, which combine physical demands with significant cognitive challenges, produce more potent effects on executive functions compared to more traditional exercise protocols. Similarly, the heterogeneity for cognitive flexibility was largely driven by a single study, Silva et al. (2020). The key distinguishing characteristic of this trial was its exclusive recruitment of children with Attention Deficit Hyperactivity Disorder (ADHD). The response to exercise interventions in populations with specific neurodevelopmental disorders may differ significantly from that of typically developing adolescents, likely accounting for this study’s outlier status. Taken together, these sensitivity analyses not only confirm that the overall positive effects of exercise on these domains are robust, but also highlight that specific intervention types and participant characteristics are crucial moderators that can explain the inconsistent results across the literature.”

The study primarily focused on participants aged 8–18 years, encompassing both elementary and middle school students. Thus, it can be concluded that children in the elementary age group also derive cognitive benefits from physical activity. This may be attributed to the game-oriented nature of elementary-aged children, who develop specific cognitive skills through play and physical activities (Leppo et al., 2000), thereby enhancing their overall cognitive functions. Middle and high school students also derive benefits; however, their executive function scores tend to decline with age (Fedewa and Ahn, 2011). Nonetheless, further research is required to elucidate the specific mechanisms underlying these findings. For instance, longitudinal studies could provide insights into the moderating effects of age and grade on the relationship between physical exercise and cognitive outcomes.

This study incorporated a paper focusing on children with ADHD (Silva et al., 2020). ADHD, a neurodevelopmental disorder, is characterized by symptoms of inattention, hyperactivity, and impulsive behavior. ADHD does not directly impair children’s intelligence or learning abilities; however, it can indirectly influence academic performance by disrupting attention, behavioral self-regulation, and organizational skills. A previous narrative review (Bluechardt et al., 1995) suggested that children with learning disabilities may not experience cognitive benefits from physical exercise. However, the findings of this study suggest that physical activity is equally beneficial for children with ADHD as it is for typically developing children. Moreover, physical activity could serve as an important component of educational programs designed for children with learning disabilities.

Our findings suggest that the type of physical exercise is not a significant determinant of cognitive development. Rather, engaging in any form of physical activity ultimately enhances cognitive performance. The literature reviewed includes various forms of exercise, such as aerobic exercises, endurance training, strength training, and coordination exercises. All these types of physical activity contribute to enhancing cognitive function in children and adolescents. However, our subgroup analysis revealed that aerobic exercise had the most significant impact on cognitive function compared to other exercise modalities. This finding aligns with a previous meta-analytic study, which concluded that all forms of physical exercise can enhance cognitive abilities (Sibley and Etnier, 2003). However, different forms of exercise may exert varying effects on the development of specific aspects of cognitive abilities. However, due to the relatively small number of studies included in this meta-analysis, as well as the high heterogeneity and small sample sizes in some studies, the findings should be interpreted with caution. Additionally, since various exercise modalities were examined, our subgroup analysis revealed that aerobic exercise had the most significant impact on adolescents’ cognitive function. Future research should focus on identifying which specific exercise types are most effective in enhancing cognitive function in adolescents, particularly examining the differential effects of aerobic exercise compared to other exercise modalities. High-quality studies with larger sample sizes and more consistent exercise protocols are needed to confirm these findings. Furthermore, cultural differences across the studies may have influenced the results, and the long-term effects of exercise on cognitive function remain uncertain, as most studies have focused on short-term outcomes. These factors should be addressed in future research to provide a more comprehensive understanding of the effects of physical exercise on cognitive function.

Fedewa and colleagues (Fedewa and Ahn, 2011) suggested that aerobic exercise is more effective in promoting cognitive function development compared to perceptual-motor and stretching exercises. Previous studies have identified aerobic exercise as the gold standard for enhancing cognitive function (Etnier et al., 2006). However, recent research suggests that sports and exercises involving cognitively demanding tasks (e.g., soccer, basketball, tennis) can significantly enhance executive function. For instance, Tsai et al.(Tsai et al., 2012) implemented a 10-week soccer training program incorporating cognitively demanding tasks for children with Developmental Coordination Disorder (DCD). Through these exercises, children with DCD actively engaged in neuroresponsive processes linked to executive function, which contributed to improvements in their executive function levels.

Our study suggests that physical exercise enhances cognitive function in adolescents, primarily through brain-related changes, such as increased cerebral blood flow and improved coordination between the cerebellum and prefrontal cortex, which collectively elevate cognitive performance in children and adolescents (Gligoroska and Manchevska, 2012; Chaddock-Heyman et al., 2013). Physiological responses triggered by physical exercise, including changes in heart rate (Kamijo et al., 2004), brain-derived neurotrophic factor (BDNF) levels (Ferris et al., 2007), and plasma catecholamine levels (Chmura et al., 1994), have been shown to influence cognitive function. These physiological changes, along with increased cerebral blood flow and dopamine levels, contribute to enhanced cognitive function. They further improve executive function, attention, working memory, cognitive flexibility, and inhibitory control in children and adolescents through physical exercise (Sung et al., 2022). Physical exercise interventions enhance cognitive function in children and adolescents, with these improvements observable at the systemic, molecular, and cellular levels (Ratey and Loehr, 2011). From a systems perspective, electrophysiological and neuroimaging studies suggest that physical exercise may enhance the efficiency and adaptability of the nervous system in domains such as attention, learning, and memory (Ratey and Loehr, 2011; Morgan et al., 2015). Such improvements in neural efficiency are thought to contribute to reduced response times and faster reactions (Ratey and Loehr, 2011). At the molecular level, physical exercise is believed to increase the availability of neurotrophins and growth factors. These molecules are known to regulate cellular processes that support brain plasticity, including synaptic plasticity, neurogenesis, and angiogenesis (Phillips et al., 2014). One such mechanism, cerebral blood flow, can be monitored using functional near-infrared spectroscopy (fNIRS). Because fNIRS is non-invasive and does not require specific postures or controlled environments like functional magnetic resonance imaging (fMRI), it is widely utilized in brain imaging research with children (Moriguchi and Hiraki, 2013). Emily Bremer (Bremer et al., 2020) and colleagues conducted a controlled trial and found that 20 min of physical exercise significantly increased task-related brain blood flow in the prefrontal cortex of male children compared to their sedentary peers, leading to improved cognitive function. While our study suggests that physical exercise enhances cognitive function in adolescents through brain-related changes, such as increased cerebral blood flow and improved coordination between the cerebellum and prefrontal cortex, it is important to note that causality cannot be conclusively determined in this analysis. Although we observed correlations between physical exercise and cognitive improvements, the exact mechanisms behind these effects remain uncertain. Future research should delve deeper into understanding how exercise-induced physiological changes, such as neurotrophic factor levels, dopamine regulation, and brain blood flow, directly contribute to cognitive enhancement. Additionally, exploring how exercise protocols influence these mechanisms over the long term will help clarify the enduring effects of exercise on adolescent cognitive development.

Physical exercise positively influences the mental health of adolescents, including reductions in depression and anxiety (Parfitt and Eston, 2005). Furthermore, both direct and indirect physiological, cognitive, emotional, and learning mechanisms link engagement in physical activities and high levels of physical fitness to enhanced brain structure, brain function, and cognitive performance (Hillman et al., 2008). Physical exercise not only enhances cognitive function in adolescents but also positively influences their academic performance (Hatch et al., 2021). Physical exercise may also positively impact adolescents’ mental health, including reductions in depression and anxiety (Parfitt and Eston, 2005). Furthermore, both direct and indirect physiological, cognitive, emotional, and learning mechanisms link participation in physical activities and high levels of physical fitness to improved brain structure, brain function, and cognitive performance (Hillman et al., 2008). Physical exercise not only enhances cognitive function in adolescents but also positively impacts their academic performance (Hatch et al., 2021). Contemporary researchers suggest that the impact of exercise on children’s academic performance is mediated by improvements in cognitive domains, including executive function, memory, and fluid intelligence (Tomporowski et al., 2015). Participation in physical exercise creates essential pathways and experiences that support the healthy development of cognitive abilities (Sung et al., 2022). When children participate in various stimulating activities, they must focus their attention, retain instructions, and frequently suppress irrelevant actions in their environment to successfully complete specific tasks or activities. Similarly, participation in physical exercise involves the use of executive functions, including inhibitory control, decision-making, and processing speed, to enhance athletic performance (Tsai et al., 2012). These learning processes may inherently enhance cognitive function. Thus, incorporating moderate-intensity physical exercise into school programs not only supports the improvement of adolescents’ cognitive function but also fosters their academic performance. For instance, Mahar et al. (2006) and Erwin et al. (2012) implemented in-class interventions aimed at improving children’s attention and observed subsequent enhancements in their academic performance.

Our research indicates that physical exercise enhances adolescents’ executive function, attention, working memory, cognitive flexibility, and inhibitory control. However, the effects of physical exercise on cognitive abilities vary depending on the intensity, frequency, and type of exercise. Nonetheless, all studies support the notion that increased participation in physical exercise and longer exercise durations are associated with improved cognitive outcomes(Best, 2010). To date, the majority of studies have focused on aerobic exercise (i.e., sustained moderate- or low-intensity activity) to investigate the effects of physical exercise interventions or training on brain function(De Diego-Moreno et al., 2022). In certain controlled trial analyses of acute and chronic exercise, modest improvements were observed in attention, processing speed, and working memory (Smith et al., 2010). Exercise intensity is thought to affect cognitive performance in an inverted U-shaped manner (Brisswalter et al., 2002). Researchers hypothesize that cognitive function improves as exercise intensity increases, up to a critical threshold, beyond which it begins to decline. However, some studies have not confirmed that high-intensity or overload exercise negatively impacts cognitive function. For instance, in complex tasks like decision-making, significant improvements were observed at both moderate and high intensities, with effect sizes of −0.8 and −0.6, respectively (Brisswalter et al., 2002). A meta-analysis demonstrated that acute high-intensity exercise produces a small yet significant improvement in post-exercise executive function. The meta-analytic estimate of this effect is d = 0.24, 95% CI [0.13, 0.35](Moreau and Chou, 2019). Although findings on the effects of high-intensity or overload exercise remain inconclusive, its impact on cognition has been reported to be both positive (Moreau and Chou, 2019) and negative (Smith et al., 2016). Scientific evidence indicates that moderate-intensity aerobic exercise positively influences cognitive function in children and adolescents (Lambourne and Tomporowski, 2010). A recent meta-analysis found that moderate-intensity aerobic exercise results in greater improvements in cognitive abilities compared to both light- and high-intensity exercise, further supporting this theory (McMorris and Hale, 2012).

International recommendations from the World Health Organization(Okely et al., 2021) and the 2018 U.S. guidelines(King et al., 2019) advise that adolescents engage in at least 60 min of moderate-to-vigorous physical activity daily. A 2020 study by Guthold et al., published in The Lancet, reported that over 80% of adolescents aged 11–17 worldwide fail to meet the recommended daily levels of physical activity, adversely affecting both their current and future health (Guthold et al., 2020). Therefore, increasing the frequency of physical exercise among adolescents is crucial, as physical activity plays an indispensable role in their development by enhancing overall fitness and supporting cognitive function. These health benefits often persist from childhood into adulthood, contributing to the prevention of cognitive dysfunction (Hayes et al., 2019).

This study adhered to the PRISMA guidelines; however, several shortcomings and limitations remain. First, the literature search did not include unpublished studies, and some studies were excluded due to incomplete outcome data, potentially impacting the comprehensiveness of the data. Additionally, the relatively small sample size of the Meta-analysis may have reduced the reliability of the results. Lastly, although three researchers independently conducted a double-blind quality assessment of the included studies, the evaluation relied solely on the “Cochrane Risk of Bias Tool.” This reliance, combined with the possibility of subjective judgment errors, may have introduced specific biases. To address this, incorporating additional evaluation criteria is recommended to minimize individual assessment bias.

## 5 Conclusion

This systematic review and meta-analysis demonstrates that regular exercise significantly benefits the cognitive development of children and adolescents, particularly in areas such as executive function, attention, inhibitory control, working memory, and cognitive flexibility. The subgroup analysis reveals that aerobic exercise has the greatest impact on adolescents’ cognitive function. Thus, exercise serves as an effective strategy to promote cognitive development in adolescents and enhance their academic performance.

## Data Availability

The original contributions presented in the study are included in the article/supplementary material, further inquiries can be directed to the corresponding author.

## Footnotes

^1^ https://www.crd.york.ac.uk/prospero/
